# Nanomechanics in Monitoring the Effectiveness of Drugs Targeting the Cancer Cell Cytoskeleton

**DOI:** 10.3390/ijms21228786

**Published:** 2020-11-20

**Authors:** Andrzej Kubiak, Tomasz Zieliński, Joanna Pabijan, Małgorzata Lekka

**Affiliations:** Department of Biophysical Microstructures, Institute of Nuclear Physics, Polish Academy of Sciences, PL-31342 Kraków, Poland; Andrzej.Kubiak@ifj.edu.pl (A.K.); Tomasz.Zielinski@ifj.edu.pl (T.Z.); Joanna.Pabijan@ifj.edu.pl (J.P.)

**Keywords:** atomic force microscopy, cell biomechanics, cell cytoskeleton, antitumor drugs, monitoring drug efficiency

## Abstract

Increasing attention is devoted to the use of nanomechanics as a marker of various pathologies. Atomic force microscopy (AFM) is one of the techniques that could be applied to quantify the nanomechanical properties of living cells with a high spatial resolution. Thus, AFM offers the possibility to trace changes in the reorganization of the cytoskeleton in living cells. Impairments in the structure, organization, and functioning of two main cytoskeletal components, namely, actin filaments and microtubules, cause severe effects, leading to cell death. That is why these cytoskeletal components are targets for antitumor therapy. This review intends to describe the gathered knowledge on the capability of AFM to trace the alterations in the nanomechanical properties of living cells induced by the action of antitumor drugs that could translate into their effectiveness.

## 1. Introduction

Atomic force microscopy (AFM) is mainly recognized as a technique applied to visualize surface topography with nano- or subnanometer resolutions. Currently, with the gathered research data, it has been shown that AFM can be successfully employed in the nanoscale measurements of biological samples, such as proteins or living cells [1,2,3]. Compared to other high-resolution techniques such as scanning/transmission/cryo-electron microscopy, the essential advantage of AFM is the capability to measure the biophysical properties of biological samples in physiologically relevant conditions. Till now, this technique has been widely applied to study the nanomechanical properties of healthy and pathologically altered cells and tissues; those elastic properties were quantified through an elastic (Young’s) modulus [4,5,6,7,8,9]. In one of the first papers, it has been demonstrated that AFM-based nanomechanical characterization can be applied to cancer cells. The results revealed larger deformability of bladder cancer cells than non-malignant cells (a larger deformability denotes a smaller Young’s modulus). A significantly lower Young’s modulus was correlated with a poorly differentiated cell cytoskeleton [3]. At present, it is well-established that cancer progression manifests or induces the alterations in cell deformability in the majority of cancers, including thyroid [4], ovarian [10], breast [11,12,13,14], prostate [15], bladder [16,17], and pancreas [18]. Moreover, for some cancers like ovarian cancer [10] and melanoma [19], it is possible to correlate larger cellular deformability with cancerous cells’ invasiveness.

Apart from identifying the pathologically altered cells by their biomechanical properties, part of the current research showed successful use of AFM to evaluate changes in individual cells induced by the action of antitumor drugs. The presented review aims to summarize the existing knowledge and simultaneously underlie the advantages of AFM usage to identify the mechanisms or quantify the antitumor drugs’ effectiveness based on a nanomechanical approach.

## 2. Biomechanical Properties of Cells Measured by AFM

### 2.1. Brief Description of AFM-Based Elasticity Measurements

**Figure 1:**
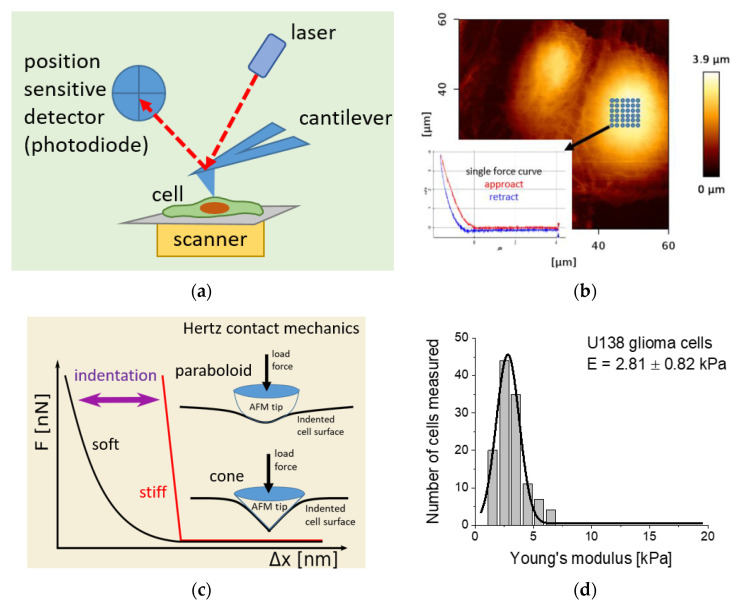
(**a**) Illustration of the main elements constituting an atomic force microscope (AFM). (**b**) In the AFM-based elasticity measurements, a grid is placed over a central part of the cell. At each point, a so-called force curve is recorded (inset). (**c**) In nanomechanical analysis, the approach part of the calibration force curve (reference, curve acquired on a stiff, non-deformable surface) is subtracted from that measured on a cell. The obtained relation between the force and indentation is further used to calculate Young’s (elastic) modulus by applying Hertz contact mechanics with Sneddon modifications approximating the geometry of the probing tip. Typically, either a paraboloidal or a conical shape of the indenting AFM tip is considered. (**d**) The final Young’s modulus is frequently derived from a histogram by fitting Gauss or lognormal functions (exemplary results obtained for U138 glioma cells analyzed with Hertz–Sneddon mechanics, assuming a conical shape of the probing AFM tip).

In the atomic force microscope, a sample surface is probed by a delicate cantilever, being a flat, rectangular, or triangular spring possessing a probing tip at the free end. The cantilever is moved in a raster scan in proximity to the sample surface. The interaction forces acting between the tip and the sample surface cause the deflection of the cantilever. Such a deflection is typically detected by an optical system consisting of a laser and position-sensitive photodetector (a photodiode). In this detection system, a laser beam is focused on the free end of the cantilever. The reflected beam is recorded by a photodiode whose active area is divided into four quadrants (Figure 1a).

Cantilever deflection perpendicular to the sample surface reflects the surface topography that corresponds to the laser beam’s displacements between the upper and bottom quadrants. In parallel, the cantilever’s twist caused by the friction forces is recorded as the difference between the left and right quadrants. AFM operates in various conditions, including vacuum, ambient, or liquids. The latter enables us to measure biological objects in a physiologically relevant environment. One of AFM’s indispensable advantages is its ability to quantify the sample’s physicochemical properties; thus, to deliver information on its elasticity, viscoelasticity, and adhesiveness [20,21]. For such measurements, the force spectroscopy mode is employed. Here, the cantilever is located over a central part of the cell where a grid is set (Figure 1b). At each point of the grid, the cantilever is moved towards the sample surface, brought in contact, and then withdrawn. During such a cycle, a single force curve is recorded (inset in Figure 1b).

### 2.2. Hertz–Sneddon Contact Mechanics in Determination of Mechanical Properties of Cells

The biomechanical properties of cells can be characterized by various measures, such as elasticity (elastic modulus), viscoelasticity, and stiffness. All of them describe the behaviour and/or response of a cell to the applied deformation (strain is the amount of deformation); however, each of them describes a specific mechanical component. Focusing on elastic properties, elasticity describes the material deformed reversibly by the external load (stress). When force is removed, the deformations are fully reversed. For ideally elastic material, the stress–strain relationship (referred to here as the force–deformation relation) is linear. The biomechanical properties of a specific material can be quantified either as a stiffness or elasticity. The former denotes the resistance of a solid body to deformation caused by an external force whereas the former is an intrinsic property of the material. Stiffness depends on the solid body size, shape, amount of the material, and boundary conditions, whereas elasticity not. Although stiffness and elasticity cannot be directly compared, they are qualitatively related; namely, a low stiffness denotes a low elastic modulus.

In the majority of AFM-related studies, the elastic (Young’s) modulus is used as a semi-quantitative measure of the living cell’s biomechanical properties [6]. The semi-quantitative character of the modulus stems mainly from several factors: the indentation depth is not measured; the load force is not controlled—only deflection can be controlled in the AFM; the pyramidal geometry of the probing AFM tip is approximated by a cone or paraboloid; the cells are not ideally elastic materials, but they reveal viscoelastic and time-dependent behaviours; and the cells are not homogenous as they have an internal structure composed of blocks of various mechanical properties. Due to the relativeness of the AFM-derived elastic modulus, it can be used only for comparative analysis under the requirement that all experimental conditions are preserved for all investigated samples.

Young’s modulus is derived based on the analysis of the force curve’s approach. Each recorded force curve represents the relation between the cantilever deflection and a relative scanner (or sample or tip) position. Cantilever deflection is converted into the load force by multiplying by the cantilever spring constant and photodetector sensitivity [6]. It must be underlined here that, in AFM, the indentation depth is not measured. To obtain the relation between the load force and the indentation depth, the reference curve, i.e., force curve recorded on a stiff, non-deformable surface like glass or a Petri dish, is subtracted from the force curve recorded on a cell (Figure 1c). The resulting load force–indentation depth curve is fitted with the equation describing contact mechanics [22,23,24,25]. The apparent, average Young’s (elastic) modulus is determined from the Gaussian or lognormal fit to the modulus histogram (Figure 1d).

The nanomechanical analysis applied to the data recorded by AFM, typically uses the Hertz contact mechanics with Sneddon modifications (referred to here as the Hertz–Sneddon contact mechanics, [22,23]). The Hertz mechanical model was developed to describe the relationship between the load and indentation of two purely elastic spheres pressed against each other with a load force [22]. The model could be extended into the case of a rigid sphere indenting a purely elastic flat half-space by setting the radius of one sphere to infinity. The use of the Hertz contact mechanics implies several assumptions being not entirely fulfilled in the case of the mechanical properties of the living cells [13,24,25]. Among them, the most essential are the lack of adhesion and friction within the contact area, limitation to homogenous and isotropic materials, assumption of a spherical contact region, and small deformations (a range of indentation limited by the diameter of the probing sphere) located within the elastic limit. Moreover, the indentation depth should not exceed ca. 10% of the sample thickness. The contribution of various indenter geometries to the relation between the load and indentation was resolved by solving the Boussinesq problem, i.e., finding the elastic state in a linearly elastic isotropic half-space, subjected to a perpendicular load applied in a point of its boundary plane (Sneddon contact mechanics, [23]). Thus, the AFM probes that are often four (or three)-sided pyramids can be approximated by a cone or paraboloid. Less frequently, the Oliver–Pharr model has been used to quantify the elastic modulus from the retraction part of the force curve recorded for samples, for which the indentation caused permanent, plastic deformation [24]. The limitations of these models are a lack of knowledge on the contact surface area of the indenter and lack of permanent, plastic deformation induced by the AFM tip indenting the living cells. It should be noted here that adhesion and friction are considered neither Hertz nor Sneddon nor Oliver–Pharr contact mechanics.

Adhesion occurring between the indenter and the sample surface was introduced in the 1970s by the Johnson–Kendall–Roberts (JKR, [26]) and Dejarguin–Muller–Toporov (DMT, [27]) theoretical models. Both models derive the relationship between the force and indentation from the balance between adhesion and elasticity. Adhesive forces foster the contact being the non-negligible contribution to the elastic reaction force, causing the material deformation. In the JKR model, the adhesion is considered to be present inside the contact area, while the DMT model ponders the adhesion as a long-range interaction present outside the contact area. The JKR model is valid in the case of strong adhesion between the indenter and soft samples and for indentations smaller than the radius of the indenting sphere. Oppositely, DMT is valid for stiffer samples with weaker adhesion. These models are considered as two extreme limits and have opened the path to develop a unified theory describing better the interplay between a material’s adhesion and elasticity.

## 3. Nanomechanical Properties of a Cell Cytoskeleton

**Figure 2:**
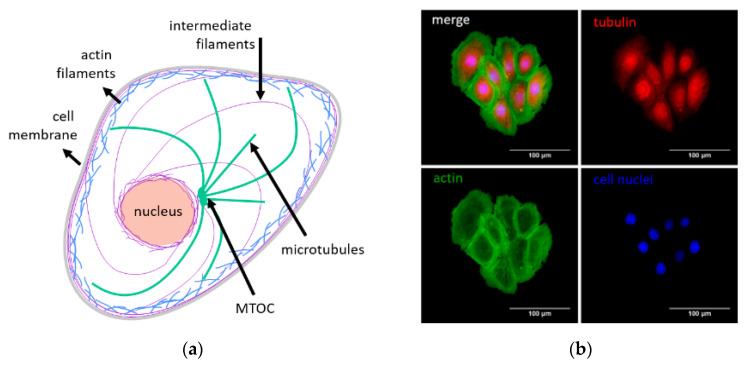
(**a**) A scheme showing the organization of three main cytoskeletal elements (actin and intermediate filaments, microtubules) inside the cell in relation to the cell membrane and nucleus (MTOC–a microtubule-organizing center). (**b)** Exemplary fluorescent images were collected for DU145 prostate cancer cells showing labeled actin filaments and microtubules.

The cytoskeleton is one of the main cellular structures responsible for maintaining cell mechanical integrity and resistance [28]. It is a dynamic network forming a scaffold inside the cell. It is composed of three main fibrillary structures, namely, microtubules, actin, and intermediate filaments (Figure 2a,b).

Actin filaments are composed of F-actin forming thin, polar fibers with a diameter of about 7 nm. They create a mesh built up of short filaments (so-called actin cortex) localized beneath the cell membrane. The other structures made of F-actin are long and thick fibers spanning over a whole-cell volume (so-called stress fibers). An individual microtubule is formed from laterally bounded protofilaments. Each protofilament consists of interconnected αβ-tubulin dimers [29]. Microtubules spread radially from a microtubule-organizing center (MTOC) located near the nucleus to be anchored in the cell membrane. Apart from being a structural element, microtubules participate in various processes, such as cell division [30,31], cell migration [32], linkage between transmembrane and cytoplasmic proteins [33,34], transport of membrane vesicles or other organelles inside the cell, and/or facilitating the turnover of adhesion plaques [35,36,37].

With the rising attention of AFM’s employment in studies of living cells’ nanomechanical properties, an interest in anti-cytoskeletal agents occurred [38,39,40]. Due to their strong interaction with the cytoskeleton, which impairs essential cell processes such as the adhesion and migration of cells, these compounds became in focus for various direct and combined antitumor therapies. In the AFM field, these agents are typically applied to verify the participation of both actin filaments and microtubules in cell mechanics.

**Figure 3:**
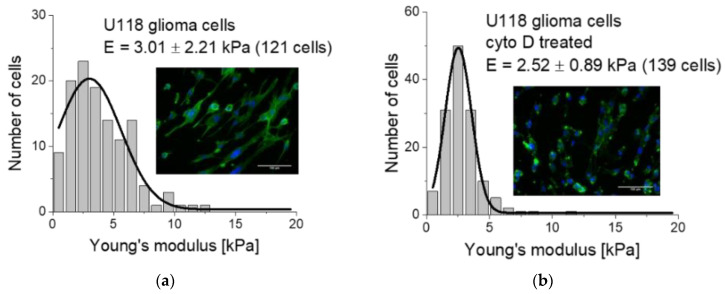
Nanomechanical properties of glioma U118 cells (**a**) before and (**b**) after the treatment with cytochalasin D (cyto D, 5 µg/mL, 10 min). Cytochalasin D induces a softening of the cells linked with reorganization (depolymerization) of the actin filaments. Insets: Images showing actin filaments stained fluorescently with phalloidin Alexa Fluor 488 dye.

The most common agents affecting actin filaments’ organization belong to the cytochalasin family, encompassing several toxins. Members such as cytochalasin B and cytochalasin D are applied to disrupt F-actin, a polymerized form of actin [41,42]. A low Young’s modulus value characterizes the observed increase in cell deformability. Due to the F-actin disruption, a cell becomes more homogenous in terms of mechanical properties, observed as a narrow elastic modulus distribution. Exemplary data showing the nanomechanical properties of U118 glioma cells treated with cytochalasin D are presented in Figure 3a,b.

A majority of the gathered data demonstrated that the nanomechanical measurements of living cells reveal the alterations in the actin network. This was confirmed by the experiments, in which cytochalasins, jasplakinolide, or latrunculin A were applied to cells [13,14,16,17,38]. Thus, it is believed that nanomechanical changes contain a dominant component originating from actin filaments; however, this feature seems to be cell-type dependent. To assess whether the nanomechanical properties of cells are sensitive to a reduction in F-actin and/or microtubule organization, a combination of drugs such as latrunculin B and nocodazole could be applied to depolymerize the actin filaments or to impair the polymerization of the microtubules. In an exemplary study, the result of such a treatment has shown a dose-dependent deformability of NIH/3T3 fibroblasts [43].

## 4. Mechanics of Actin Filaments and Microtubules as Targets for Antitumor Therapy

During cancer progression, cells adjust their structure through the reorganization of actin filaments and microtubules. Therefore, these elements seem to be a perfect target for antitumor drugs due to their importance in maintaining vital cell functions like cell adhesion or migration [44]. The disruption of both actin filaments [45] and microtubules [46] can induce apoptosis, being the final effect of antitumor drugs desired by medical doctors and patients. Although this phenomenon, the use of actin filaments as targets for antitumor therapy is highly demanding as compared to microtubules because of their strong participation in multiple cellular processes. Therefore, the research taken on actin mechanics delivers better insights into the mechanisms involved in various cellular processes than revealing the effect of the antitumor drugs [47,48,49,50]. Cancerous cells’ ability to proliferate infinitely is characterized by fast mitotic divisions, in which microtubules participate actively [46]. That is why microtubules, or more specifically α/β tubulin heterodimers, had become targets for microtubule-targeted drugs (MTDs) used in various antitumor therapies [51]. MTDs are divided into three main classes: taxanes, vinca alkaloids, and those binding to the colchicine binding site. Depending on the tubulin dimer’s binding site locations, a diverse effect on the microtubules’ organization is observed [52,53,54,55,56]. Vinca alkaloids destabilize microtubules by binding to the site located at the inter-dimeric interface between α and β tubulin heterodimers [54]. Taxanes stabilize microtubules and simultaneously enhance the polymerization process by reversibly binding to β-tubulin at the binding site located at the microtubules’ interior lumen [54]. Colchicine blocks the availability of α/β tubulin heterodimers for protofilaments or MTs by changing the dimer conformation [57].

Among these MTDs families, currently, taxanes and vinca alkaloids are mostly applied in the treatments of various cancers [51,58,59]. Taxanes have already been extensively studied to show microtubule-related changes in the mechanical properties of cancer cells [38,60,61,62,63,64]. Various concentrations and incubation times have been applied, ranging from nM to µM and from minutes to hours or even days [39,64,65]. A summary of the nanomechanical characterization of cancer cells treated with MTDs is presented in Table 1 (the increase in Young’s modulus indicates augmented cell rigidity while its decrease reveals larger cell deformability).

The obtained results on cancer cells’ nanomechanical properties permit insight into various aspects of the taxanes’ interaction with microtubules and their effect on the microtubular network and cell behavior [60,62,63,64,69]. In most studies, AFM is also used to evaluate the morphological changes in single cells. The overall cell morphology can be quantified by various parameters, such as the surface area of the spreading cells, cell height, and membrane corrugations. Cell surface area and height are obvious morphological parameters. Using AFM, the corrugations of the cell membrane can be assessed in qualitative (images of cell membrane) and quantitative (determination of roughness value) manners. A higher roughness denotes larger membrane corrugations. For example, such changes have been reported for human lung adenocarcinoma cells (ASTC-a-1 cell line) as well as for Ishikawa and HeLa cells treated with taxol or paclitaxel, respectively [60,63]. Increased height or altered surface roughness of these cells seem to be an early sign of apoptosis accompanied by cell softening or stiffening. The process is dependent on various factors, including cell and taxane types, dose, and time. For HeLa cells treated with paclitaxel, the deformability decreases within a time frame of 6–12 h (cell become rigid), followed by increased cell deformability (cells become compliant) for a more prolonged treatment time. Thus, changes in cell deformability can be correlated with the paclitaxel-activated apoptosis [63].

To enhance the therapy, clinicians are frequently combining taxanes with other antitumor drugs, often with the members of the vinca alkaloids family such as vincristine or vinorelbine. Such a combination of drugs has a twofold effect on cells—taxanes stabilize the microtubular network. At the same time, vinca alkaloids destabilize. However, consequently, they lead to impairments in the dynamics of the microtubular system and induce apoptosis. Taxanes and vinca alkaloids are not the only pair of microtubule-targeted agents studied. The other combinations employed are taxol/colchicine [70] or paclitaxel/S-HM_-3_ (a tumor angiogenesis inhibitor with a short half-life) [71]. Analogously to vincristine, colchicine destabilizes microtubules. The cells from a pro-monocytic, human myeloid leukemia cell line (U937 cell line) treated with these drugs change the mechanical properties in a drug-type-dependent manner. For small indentations, the colchicine-treated cells exhibited a larger deformability (low Young’s modulus), which decreased in the taxol-treated cells (cells become rigid). As taxol induces microtubule assembly, stiffening of cells is expected. On the contrary, colchicine-induced microtubule disassembly should manifest in the increased deformability of the cells (a cell softening). The differences in the nanomechanical properties of the taxol-treated and the colchicine-treated cells give evidence that microtubules strongly participate in the nanomechanical stability of the cells.

## 5. Non-Cytoskeleton Interacting Drugs Affecting Cancer Cell Biomechanics

The high sensitivity of AFM in measurements of living cells’ nanomechanical properties gives rise to whether only the effects of antitumor drugs interacting with the cell cytoskeleton are possible to detect. In several already published research papers, changes in the nanomechanical properties have been recorded in cases where the applied drugs were not interacting directly with the cell cytoskeleton. In one of the first papers, chitosan’s influence on human bladder cells’ mechanical properties were studied [16]. Chitosan is a linear polysaccharide derived from chitin. It has a potential antitumor action by inhibiting the glycolytic activity of cancer cells. Cells treated with a microcrystalline chitosan with three different deacetylation degrees show no changes in cells from non-malignant cell cancer of the ureter (HCV29 cell line). In contrast, cells from transitional cell carcinoma (T24) were characterized by a significant drop in cell deformability (Young’s modulus increased). Changes in the mechanical properties were associated with alterations in the cells’ glycolytic activity. Although chitosan is not interacting directly with cytoskeletal elements, changes in the cells’ mechanical properties indicate its indirect effect through glycolytic enzymes. Some of the glycolytic enzymes are present either in the cytosol or associated with the cytoskeleton [72]. Thus, the cytoskeleton-associated enzymes’ detachment from the cytoskeleton manifests in the decrease in glycolysis level and reorganization of the cell cytoskeleton [16]. Another work has reported a study of the morphology and mechanical properties of human prostate adenocarcinoma cells (PC-3) during a binding of human endogenous antimicrobial neutrophil peptide-1 (HNP-1), being cytotoxic to cancerous cells [73,74]. The untreated PC-3 cells present an epithelial morphology with a smooth surface and the presence of pseudopodia. Upon HNP-1 addition, the morphology shows an irregular cell shape and fragmentation of the nucleus. These changes were accompanied by a larger deformability of the HNP-1-treated cells. In another example, the structural and mechanical properties of the neuroblastoma SH-SY5Y cells induced by the N-methyl-D-aspartate (NMDA) receptors (a subtype glutamate receptor) were studied in a time-dependent manner. AFM reveals the rougher surface and more rigid cells attributed to the real-time degeneration [71]. Targeting the low-density lipoprotein receptor-related protein 1 (LRP-1) is considered a promising therapeutic target as LRP-1 can internalize the proteases involved in cancer progression. A lack of LRP-1 in the cells decreases their invasive capabilities [75]. That is why strategies targeting LRP-1 could affect the capability of cancer cells to invade. The use of the AFM, conducted on the FTC-133 human thyroid carcinoma cell line, demonstrated the LRP-1 induced phenotypic changes in cancer cells. The effect of antitumor drugs on cell mechanics is presented in Table 2.

The data gathered so far reveal a systematic increase in Young’s modulus regardless of the antitumor drug applied to the cancer cells. Interestingly, these compounds, presented in Table 2, do not directly interact with the cytoskeletal elements, but they induce a biomechanical indicator of the effectiveness of their action.

An interesting class of antitumor drug that inhibits the growth of tumors, which is not considered here, applies fullerenes/fullerenes/fullerenols or various types of nanoparticles, made of, e.g., titanium dioxide (TiO_2_). These small particles internalized inside the cells produce reactive oxygen species (ROS) upon the incident visible light, causing cell apoptosis and leading to cancer inhibition [82,83,84,85]. In the abovementioned studies, AFM was applied to study the influence of potential antitumor drugs on living cells. Although they only reach surface receptors that are indirectly linked with the cell cytoskeleton, such drugs’ action reveals significant remodeling of the targeted cells’ surface and cytoskeleton, as shown from the topographical and nanomechanical measurements realized by AFM.

## 6. Biomechanics in Cancer Cell Resistance to Antitumor Drugs

During the applied antitumor therapy, the drug’s resistance after repeated treatment appears in most patients, seriously affecting the prognosis. Therefore, AFM’s capability to trace changes in the morphology and nanomechanics of cancer cells is an essential advantage used to understand how these drugs interact with the cytoskeleton or whether they can act synergistically and enhance apoptosis as a primary mechanism of cell killing [61,86].

In one of the exemplary studies on drug-resistance cells, the findings revealed that cisplatin resistance is dependent on the organization of actin filaments linked with the mechanical, morphological, and migratory properties of ovarian cancer cells [87]. Cisplatin-resistant (A2780cis) ovarian cancer cells displayed about three times higher migratory behavior than cisplatin-sensitive (A2780) cells. Young’s modulus for these cells increased (from 80 ± 49 Pa to 273 ± 236 Pa for A2780 and A2780cis cells, respectively), indicating the loss of cell deformability in the drug-resistant cells. More rigid cells can generate enough traction force to penetrate through the surrounding matrix. Thus, A2780cis cells have a better chance to move to avoid/minimize the effect of the drug treatment [88].

Further experiments conducted on nine ovarian cancer cell lines showed that cisplatin resistance scales linearly with decreasing cell deformability [89]. A study on prostate cancer is another example of the applicability of nanomechanics–based AFM measurements to monitor the effect of antitumor drugs [39]. All the drug-treated prostate cancer cells (PC-3) were more rigid as compared to the untreated cells. Such a stiffening was found to be a result of two mechanisms involved. For the three drugs, i.e., disulfiram, MK-2206 (allosteric inhibitor of a serine/threonine-specific protein kinase B (AKT)), and paclitaxel, the reconstruction of the cell cytoskeleton correlated to the stiffening of the membrane protein structure (e.g., filament shortening and thickening, with no changes in the polymerization of the actin filaments inside the cells), regardless of their mechanism of action. The other drugs, such as tomatine, BAY 11-7082 (inhibitor of κB kinase), valproic acid, 12-O-tetradecanoylphorbol-13-acetate, and celecoxib, affect not only the elasticity but also the viscosity of the PC-3 cells. In such a case, the cell cytoskeleton changes due to microtubules’ polymerization, which may cause the reorganization of the actin filaments.

It is worth mentioning that AFM measurements can detect nanomechanical changes related to chemotherapy-induced peripheral neuropathy (CIPN). One example describes the CIPN-related effect of vincristine and paclitaxel on dorsal root ganglion (DRG) neurons [61]. At a low concentration (2.5 ng/mL) of vincristine, the DRG neurons reveal little outgrowth of the neurites, stopping at higher drug concentrations. In parallel, the AFM images show a smoother surface (roughness increased by 90%). Simultaneously, nanomechanical measurements reveal increased deformability (almost two times) of the untreated DRG neurons compared to the deformability of cells treated with vincristine. In the untreated neurons, a well-organized network of microtubular networks was lost after vincristine treatment. Oppositely, paclitaxel applied to live DRG neurons showed a significant increase in Young’s modulus from 10 kPa to 18 kPa, accompanied by an intact microtubular network. By applying CIPN-activating drugs, with a distinct mechanism of action on microtubules, the evidence that there is a link between cell nanomechanics and microtubules organization was gathered.

## 7. Conclusions

Atomic force microscopy is a well-known technique in biophysical research widely applied to study and compare the mechanical properties of pathological and physiological cells and tissues [90,91]. Early studies have shown that certain diseases manifest in an altered resistance to deformation. The apparent disease is muscular dystrophy. A lack of dystrophin leads to the weakening of muscles [92,93]. Various distinct research has also indicated that altered cancer cells’ altered deformability is a manifestation of oncogenic changes [3,94]. It makes the cell cytoskeleton to be a potential target for novel anticancer treatments. Moreover, currently used therapies against cancers frequently apply to a combination of two to three established antitumor drugs [95]. Although such a treatment has been shown to be successful for patients, its main drawbacks are side effects that can be optimized by a lower-dose administration by involving nanomechanical measurements. Data gathered so far point to the stiffening of cells as the primary cellular response to the antitumor drugs’ action. In this view, the link between cell mechanics and invasiveness or migratory properties as well as the identification of drug-resistance-related mechanisms are the most significant issues to be resolved. A better understanding of how the change in cells’ mechanical properties affects the treatment’s efficacy might be beneficiary for biomedical sciences, particularly in drug design and pharmacology studies.

## Figures and Tables

**Table 1:** Mechanical properties of cancerous cells measured by AFM, treated mainly with taxol.

Drug	Cell Type	Dose Time	Elasticity Change (*E*)	Reference
Paclitaxel	prostate cancer (PC-3)	2 and 10 µM 24 h	*E* ↑	Ren et al. 2015 [39]
Docetaxel	prostate cancer (22Rv)	150 nM 24 h	*E* ↑	Raudenska et al. [66]
Docetaxel	prostate cancer (PC-3)	200 nM 24 h	*E* ↑	Raudenska et al. [66]
Paclitaxel	melanoma (B16F10)	24 nM 14 h	no change	Lin et al. [67]
Paclitaxel	melanoma (B16F10)	287 nM 30 h	*E* ↓	Lin et al. [67]
Paclitaxel	melanoma (B16F10)	20 nM 30 h	no change	Lin et al. [67]
Paclitaxel	melanoma (B16F10)	42 nM 46 h	*E* ↓	Lin et al. [67]
Paclitaxel	endometrial cancer (Ishikawa cells)	50 µM 6–18h h	*E* ↓	Kim et al. [63]
Colchicine	hepatocellular carcinoma (SMCC-7721)	0.1 µM 2h	no change	Liu et al. [68]
Colchicine	hepatocellular carcinoma (SMCC-7721)	0.1 µM 4 and 6 h	*E* ↑	Liu et al. [68]

**Table 2:** Mechanical properties of the cancer cells treated with drugs that indirectly target the cytoskeleton; their effect manifests as changes in the cell’s biomechanics.

Drug	Cell Type	Dose Time	Elasticity Change (*E*)	Reference
Chitosan	non-malignant cell cancer of ureter (HCV29)	0.05% 40 min	no effect	Lekka et al. [16]
Chitosan	transitional cell carcinoma (T24)	0.05% 40 min	*E* ↑	Lekka et al. [16]
NHP-1 (human neutrophil peptide-1)	prostate cancer (PC-3)	5 µM 4 h	*E* ↓	Gaspar et al. [73]
Disulfiram	prostate cancer (PC-3 cell line)	1 and 2 µM 24 h	*E* ↑	Ren et al. 2015 [39]
Tomatine	prostate cancer (PC-3 cell line)	1 and 3 µM 24 h	*E* ↑	Ren et al. 2015 [39]
BAY 11-7082 (inhibitor of κB kinase)	prostate cancer (PC-3 cell line)	2 and 5 µM 24 h	*E* ↑	Ren et al. 2015 [39]
Vaproic acid	prostate cancer (PC-3 cell line)	2 and 10 µM 24 h	*E* ↑	Ren et al. 2015 [39]
12-O-tetradecanoylphorbol-13-acetate	prostate cancer (PC-3 cell line)	2 and 20 µM 24 h	*E* ↑	Ren et al. 2015 [39]
Celebrex	prostate cancer (PC-3 cell line)	2 and 10 µM 24 h	*E* ↑	Ren et al. 2015 [39]
MK-2206 (allosteric inhibitor of a serine/threonine-specific protein kinase B (AKT))	prostate cancer (PC-3 cell line)	2 and 10 µM 24 h	*E* ↑	Ren et al. 2015 [39]
NMDA (N-methyl-D-aspartate receptors)	neuroblastoma (SH-SY5Y)	5 µM 1 h	*E* ↑	Fang et al. [76]
NMDA (N-methyl-D-aspartate receptors)	neuroblastoma (SH-SY5Y)	200 µM 1 h	*E* ↑	Fang et al. [76]
Cetuximab	lung cancer (A549)	20 nM 12 h	*E* ↑	Zhang et al. [77]
Resveratrol	breast cancer (MCF-7)	50 µM 3 h	*E* ↓	Iturri et al. [78]
Resveratrol	breast cancer (MCF-7)	50 µM 6 h	no change	Iturri et al. [78]
Resveratrol	breast cancer (MCF-7)	50 µM 24 h	*E* ↑	Iturri et al. [78]
Resveratrol	breast cancer (MCF-7)	50 µM 48 h	*E* ↑	Iturri et al. [78]
Cisplatin	prostate cancer (22Rv)	24 µM 24 h	*E* ↑	Raudenska et al. [66]
Cisplatin	prostate cancer (PC-3)	93 µM 24 h	*E* ↑	Raudenska et al. [66]
Disulfiram-Cu	nasopharyngeal carcinoma cells (CNE-2Z)	200 and 400 nM 6 h	*E* ↑	Yang et al. [79]
Curcumin	liver carcinoma (HepG2)	0.78 and 1.56 μg/mL 24h	*E* ↑	Olugbami et al. [80]
*K. senegalensis* hydroethanolic extract	liver carcinoma (HepG2)	25 and 50 μg/mL 24 h	*E* ↑	Olugbami et al. [80]
Dexamethasone	acute lymphoblastic leukemia (ALL)	1 µM –	*E* ↑	Lam et al. [81]
Daunorubicin	acute lymphoblastic leukemia (ALL)	1 µM –	*E* ↑	Lam et al. [81]
